# Supplementary material for: Identification of photoperiod-induced specific miRNAs in the adrenal glands of Sunite sheep (Ovis aries)
Source: Front Vet Sci. 2022 Jul 22;9:888207. doi: 10.3389/fvets.2022.888207 (PMC9354845; doi:10.3389/fvets.2022.888207)
Supplement: Supplementary file 1 [file Data_Sheet_1.ZIP › Supplementary file/miRDeep2 file.zip!/miRDeep2 file/Know_Structure/SPLP42A2/oar-mir-181a-1.pdf]

miRBase precursor : oar-mir-181a-1
Total read count : 16160
oar-miR-181a read count : 16066
remaining reads : 94

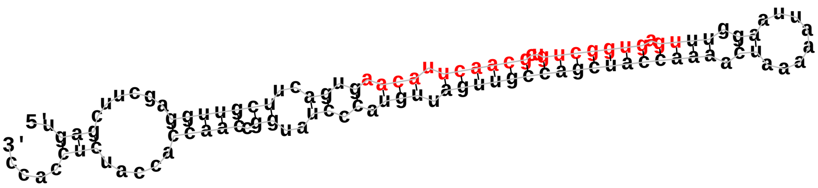

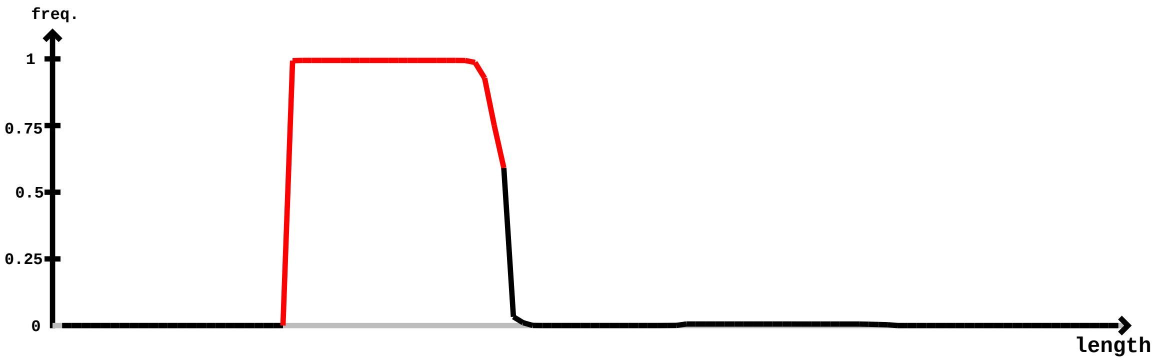

oar-miR-181a

5'- ugagcuucgagguugcuucagugaacauucaacgcugucggugaguuuggaauuaaaaaucaaaaccaucgaccguugauuguacccuauggccaaccaccaucuccacc -3'	exp		
.(((......(((((((..((.(.(((.((((((..(((((((.((((.((........)).))))))))))))))))).))).).))..)).))))).....)))....	reads	mm	sample
......................gaacauucaacgcugucggugag...	1	0	seq
.......................aacauucaacgcugucgg...	7	0	seq
.......................aacauucaacgcugucggu..	115	0	seq
.......................aacauucaacgcugucggug...	949	0	seq
.......................aacauucaacgcugucgguga..	2857	0	seq
.......................aacauucaacgcugucggugag...	2588	0	seq
.......................aacauucaacgcugucggugagu..	9006	0	seq
.......................aacauucaacgcugucggugaguu...	354	0	seq
.......................aacauucaacgcugucggugaguuu..	167	0	seq
.......................aacauucaacgcugucggugaguuug...	6	0	seq
.......................aacauucaacgcugucggugaguuugg..	1	0	seq
.......................aacauucaacgcugucggugaguuuggaauu..	1	0	seq
........................acauucaacgcugucggu..	1	0	seq
........................acauucaacgcugucggugag...	1	0	seq
........................acauucaacgcugucggugagu..	11	0	seq
........................acauucaacgcugucggugaguu...	1	0	seq
.............................caacgcugucggugagu..	1	0	seq
....uuaaaaaucaaaaccaucgaccgu..................................	1	0	seq
....aaaccaucgaccguugauugu...........................	2	0	seq
....aaaccaucgaccguugauugua..........................	2	0	seq
....aaccaucgaccguugauugu...........................	1	0	seq
....aaccaucgaccguugauugua..........................	1	0	seq
....aaccaucgaccguugauuguac.........................	2	0	seq
....aaccaucgaccguugauuguacc........................	1	0	seq
....accaucgaccguugauugu...........................	10	0	seq
....accaucgaccguugauugua..........................	11	0	seq
....accaucgaccguugauuguac.........................	15	0	seq
....accaucgaccguugauuguacc........................	47	0	seq
